# Artificial Neural Network for Response Inference of a Nonvolatile Resistance-Switch Array

**DOI:** 10.3390/mi10040219

**Published:** 2019-03-27

**Authors:** Guhyun Kim, Vladimir Kornijcuk, Dohun Kim, Inho Kim, Cheol Seong Hwang, Doo Seok Jeong

**Affiliations:** 1 Center for Electronic Materials, Korea Institute of Science and Technology, Hwarangno 14-gil 5, Seongbuk-gu, Seoul 02792, Korea; kgh920507@snu.ac.kr (G.K.); vladimir.kornijcuk@gmail.com (V.K.); galactico7@snu.ac.kr (D.K.); inhok@kist.re.kr (I.K.); 2 The Department of Materials Science and Engineering and Inter-University Semiconductor Research Center, Seoul National University, Gwanakro 1, Gwanak-gu, Seoul 08826, Korea; cheolsh@snu.ac.kr; 3 Division of Materials Science and Engineering, Hanyang University, Wangsimni-ro 222, Seongdong-gu, Seoul 04763, Korea

**Keywords:** crossbar array, artificial neural network, multilayer perceptron, resistive random access memory (RRAM), supervised learning

## Abstract

An artificial neural network was utilized in the behavior inference of a random crossbar array (10 × 9 or 28 × 27 in size) of nonvolatile binary resistance-switches (in a high resistance state (HRS) or low resistance state (LRS)) in response to a randomly applied voltage array. The employed artificial neural network was a multilayer perceptron (MLP) with leaky rectified linear units. This MLP was trained with 500,000 or 1,000,000 examples. For each example, an input vector consisted of the distribution of resistance states (HRS or LRS) over a crossbar array plus an applied voltage array. That is, for a *M* × *N* array where voltages are applied to its *M* rows, the input vector was *M* × (*N* + 1) long. The calculated (correct) current array for each random crossbar array was used as data labels for supervised learning. This attempt was successful such that the correlation coefficient between inferred and correct currents reached 0.9995 for the larger crossbar array. This result highlights MLP that leverages its versatility to capture the quantitative linkage between input and output across the highly nonlinear crossbar array.

## 1. Introduction

An artificial neural network (ANN) is a layered graph of nodes (activation units) and edges (nonzero connection weights), offering an immensely versatile hypothesis for various types of data description and different training methods [1]. Among feed-forward neural networks, multilayer perceptrons (MLP) and convolutional neural networks (CNN) are the most frequently applied types of neural network [2]. MLP is a prototypical feed-forward architecture in which every unit in a layer is fully wired to all units in the adjacent layers. In contrast, CNN has interlayer connections that are sparse and localized in the network topology [3,4]. A weight matrix in the CNN filters an input matrix fed into the next layer, and this filter (also known as convolution kernel) skims over the input layer. This is mathematically identical to convolving around the input layer, thus this architecture is termed CNN. In fact, the CNN has been successfully applied to a wide range of tasks including image recognition [1,3,4,5] and natural language processing [6].

The scope of tasks (other than conventional tasks mentioned above) within the capability of ANN has been markedly expanding, including quantum mechanical problems such as estimation of quantum mechanical ground state given a two-dimensional potential distribution [7] and modelling a mechanical system in presence of noise [8]. These examples highlight the neural network as a versatile hypothesis and the capability of backpropagation for supervised learning as a widely applicable training method.

Meanwhile, a crossbar array of nonvolatile resistance-switches, i.e., passive resistive random access memory (RRAM), ideally meets the 4F^2^ design rule (F is the minimum feature size), offering a solution to high-density nonvolatile memory [9,10,11]. Additionally, its current response to an applied voltage array naturally captures the multiply-accumulate (MAC) operation so that crossbar arrays have often been used for physical implementation of the matrix–vector product [12,13,14]. The benefit of this approach is obvious in comparison to the digital MAC operation: high speed due to the fully parallel operation and energy-efficiency due to no need for data transference during the operation. Given that the MAC operation is at the heart of MLP for both training and inference, the passive RRAM can substantially improve efficiency in MLP, which is an important field of neuromorphic engineering [12,14,15,16,17,18,19].

Considering the beneficial relationship between passive RRAM and MLP (particularly, the aforementioned passive RRAM for MLP), it is of interest to seek the reverse approach (MLP for passive RRAM). To this end, this work exemplifies the feasible application of MLP to the response inference of passive RRAM in which, once trained, the inference merely costs a few steps of matrix-vector product (depending on the depth of the network). Our new method may offer a new feasible means of crossbar circuit simulations as an alternative to conventional circuit simulation methods. 

## 2. Description of Model System

**Figure 1:**
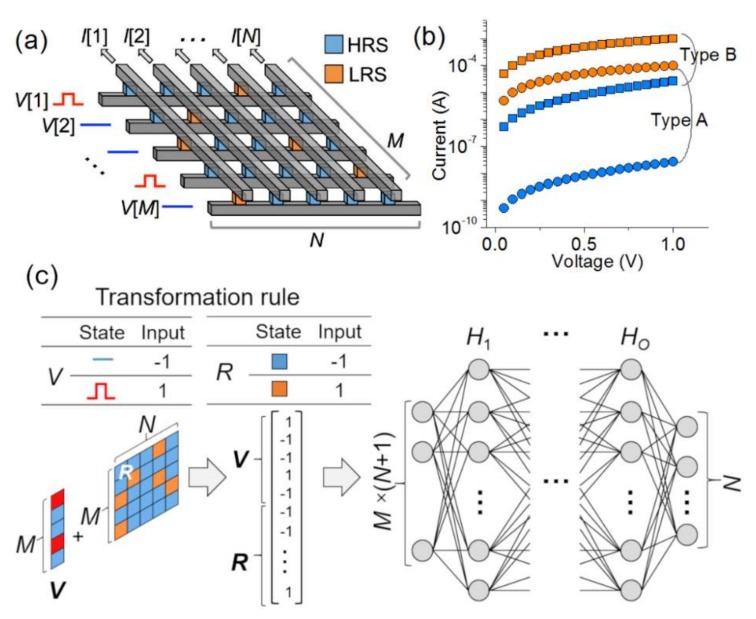
(**a**) Schematic of an *M* × *N* crossbar array. (**b**) Assumed *I*-*V* characteristics of the model resistance-switches (Types A and B). (**c**) Schematic of the MLP with *M* × (*N* + 1) input and *N* output units, and *O* hidden layers. The rule for mapping resistance-switches and input voltage arrays to an input vector is tabulated in the inset.

Passive RRAM as a model system is a *M* × *N* matrix ***R*** loaded with *R*_HRS_ and *R*_LRS_ that denote resistance in a high resistance state (HRS) and low resistance state (LRS), respectively, i.e., $R\in {\left\{R_{\mathrm{HRS}},R_{\mathrm{LRS}}\right\}}^{M\;\times \;N}$. This model system outputs an *N*-long real-valued current vector ***I*** $\left(\in {\mathbb{R}}^{N}\right)$ in response to an *M*-long real-valued input voltage vector ***V*** $\left(\in {\left\{0,1\right\}}^{M}\right)$. The model system is illustrated in Figure 1a.

The model is a nonlinear system because the HRS features a highly nonlinear current-voltage (*I*-*V*) relationship in contrast to the linear (or almost) *I*-*V* of the LRS. In this regard, the HRS was provided with a nonlinear *I*-*V* characteristic as follows: *I* = *I*_0_e*^aV^*, where *I*_0_ and *a* denote a pre-exponential factor and voltage coefficient, respectively. The larger *a*, the higher nonlinearity is given to the *I*-*V* behavior. Such nonlinearity in the HRS has been observed in an enormous number of resistance-switches given the usual thermal activation of current transport in the HRS [10,20,21]. In contrast, the LRS was given a linear *I*-*V* characteristic, keeping fidelity to experimental systems that generally represent linear or very weakly nonlinear *I*-*V* characteristics.

Two types of resistance-switch were addressed in this study: Type A and B, whose detail is tabulated in Table 1. The *I*-*V* behavior for each switch is plotted in Figure 1b. They differ in the *R*_HRS_/*R*_LRS_ ratio (evaluated at 1 V); the ratio for Type A is 100 times larger than Type B. For each type, two different array sizes (10 × 9 and 28 × 27; *M* = 10 and *N* = 9, and *M* = 28 and *N* = 27, respectively) were considered. 

## 3. Description of Artificial Neural Network

The passive RRAM outputs a current vector ***I*** that is determined by the configuration of switches over the whole array instead of their local configuration. A fully connected feed-forward network is, therefore, suitable for the model system instead of a CNN capturing patterns over local areas. Additionally, given the aforementioned nonlinearity of the model system, a hidden layer(s) needs to be incorporated in the network, rendering an MLP most suitable. Thus, an MLP was chosen as an appropriate network for the crossbar array.

Figure 1c illustrates the employed MLP with *M* × (*N* + 1) input units, *N* output activation units, and *O* hidden layers, each of which is filled with *H_i_* activation units where i ∈ {1, 2, ⋯, O}. The input into the MLP is the resistance-state (+1 and −1 for the LRS and HRS, respectively) distribution over the *M* × *N* array (***R***) plus an *M*-long vector for input voltage (+1 and −1 for *V*[*i*] = 1 and *V*[*i*] = 0, respectively) as sketched in Figure 1c. This matrix is then vectorized to feed into the MLP. The output is the estimated output current of the crossbar array at a given voltage. Note that successful training is crucial to rescale the original physical input (resistance and voltage) and output (current) in a heuristic manner such that the rescaled (scale-free) values stay in an “acceptable” range. To this end, symbolic (+1 and −1), rather than physical, values were given to the input components. Likewise, the desired (correct) output values (currents) were rescaled such that *L*[*i*] = 10 × *I*[*i*] × *R*_LRS_.

The leaky rectified linear unit (ReLU) was deployed as an activation unit: *f*(*x*) = max(*x*, 0.1*x*). The leakage when *x* < 0 is required for the negative input components. Otherwise, the negative input components are merely ignored as for the simple ReLU, *f*(*x*) = max(*x*, 0). The ReLU is a workaround for the notorious vanishing gradient problem, which is significant when the network is deep.

## 4. Training and Test Datasets

The output ***I*** in response to an input ***V*** for a given ***R*** was evaluated by applying the Kirchhoff’s circuit law to each switch. The obtained nonlinear equations were solved using the Newton-Raphson method, which resulted in the output ***I***. The calculation was elaborated in [22]. A training dataset was produced by randomly sampling resistance state distribution over the array and input ***V***. First, *p*_1_ (0 ≤ *p*_1_ ≤ 1) was randomly sampled from a uniform probability distribution function (PDF) and used as the probability that *V*[*i*] = 1. That is, if *p*_1_ is 0.4, 40% of all input lines are pulled high (1 V), and the rest lines (60%) are pulled down (0 V). Another number *p*_2_ (0 ≤ *p*_2_ ≤ 1) was subsequently sampled for each input line from a uniform PDF to randomly distribute 1 V signals over all input lines at a probability of *p*_1_ such that, when *p*_2_ ≤ *p*_1_, *V*[*i*] = 1, and 0 otherwise. This process was repeated with different *p*_2_’s over *M* rows, resulting in an input ***V*** for this training example. A third number *p*_3_ (0 ≤ *p*_3_ ≤ 1) was picked from a uniform PDF and taken as the percentage of LRS switches in the entire array. For each switch in the array, *p*_3_ was compared with another random number, *p*_4_ (0 ≤ *p*_4_ ≤ 1) was sampled for each switch, and *R*[*i*,*j*] = *R*_LRS_ when *p*_4_ ≤ *p*_3_, and *R*[*i*,*j*] = *R*_HRS_ otherwise. The label of this training example was the current response for ***I*** given ***R*** and ***V***. The complete dataset was acquired by repeating this process. The test dataset was separately made for the fair evaluation of inference accuracy.

Two different crossbar array sizes (10 × 9 and 28 × 27) for each type of switch were considered so that four different training and test datasets were produced. Each training dataset included 500,000 training examples (***V***, ***R***, and ***I***) unless otherwise specified. The network was examined for every training epoch using 10,000 test examples. Backpropagation using the mean-squared error loss function was employed with Adam optimizer that leverages learning rate adaptation for each parameter to accelerate training [23]. The MLP was batch-trained with a batch size of 100 (100 examples were randomly chosen for each training epoch). Both training and inference were performed using TensorFlow [24]. Note that for successful training, the network should vary on its hyper-parameters such as the number of ReLU units in each hidden layer (*H_i_*) and the network depth (*O*) depending on the input array length.

## 5. Training Results

**Figure 2:**
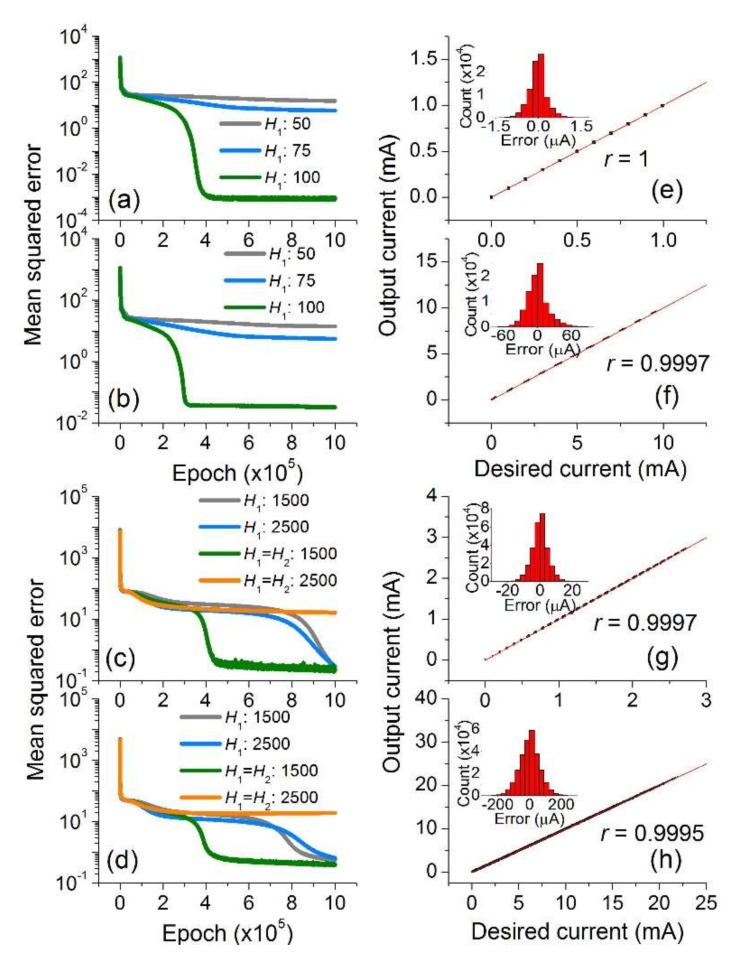
Inference-error reduction while training a network with the dataset of a 10 × 9 crossbar array of (**a**) Type A and (**b**) Type B switches. Their output results (inferred currents) for the entire 10,000 test datasets after successful training (green lines) are plotted against the desired currents in (**e**) and (**f**), respectively. The histogram of the error (the difference between inferred and desired currents) for each case is shown in the inset. The red solid lines denote the perfect match of inference with the desired (correct) results. The results are shown for a 28 × 27 crossbar array of (**c**) Type A and (**d**) Type B switches, and their statistics in (**g**) and (**h**), respectively.

Figure 2 shows a reduction in the discrepancy between the output (inferred) current ***I***_out_ and desired (correct) current ***I***_cor_ in due course, revealing successful training for all four cases conditional on the network structure. For the small crossbar array (10 × 9), a network including a single hidden layer (*O* = 1) loaded with 100 ReLU units could successfully be trained with the 500,000 training examples (Figure 2a,b). However, the use of fewer units (50 and 75) falls short of the capability of learning the dataset so that a high error level is maintained for both types of switch. This is a result of underfitting referring to the use of an unsuitable network for capturing the input pattern. Here, the network is too simple (insufficient number of units) to describe the complexity of input data.

The successfully trained network infers the output current of a random 10 × 9 crossbar array ***R*** at a random ***V***. The inferred currents for 10,000 test examples are plotted against the desired (correct) currents in Figure 2e,f, each of which includes 90,000 data points (10,000 test examples, each of which produces 9 current values). The error histogram for each case is plotted in the inset, indicating a root mean squared error (RMSE) of 0.313 μA and 17.8 μA, respectively. The larger error for Type 2 switch arises from the higher current in both HRS and LRS due to the lower *R*_HRS_ and *R*_LRS_.

The results for the larger crossbar array (28 × 27) of Types A and B switches are shown in Figure 2c,d, respectively. Given the larger input dimension (28 × 28 = 784), a network needs more units in each hidden layer and/or more hidden layers for success in training. The employed network varies on the number of units (1500 and 2500) in a hidden layer and the network depth (1 and 2). The three networks among four are given the capability to estimate the response of a random 28 × 27 crossbar array ***R*** at a random ***V***. As such, the network fully trained along the green curve for Types A and B switches represents low inference-error (a RMSE of 4.85 μA and 62.7 μA, respectively) as elucidated in Figure 2g,h, and their insets.

**Figure 3:**
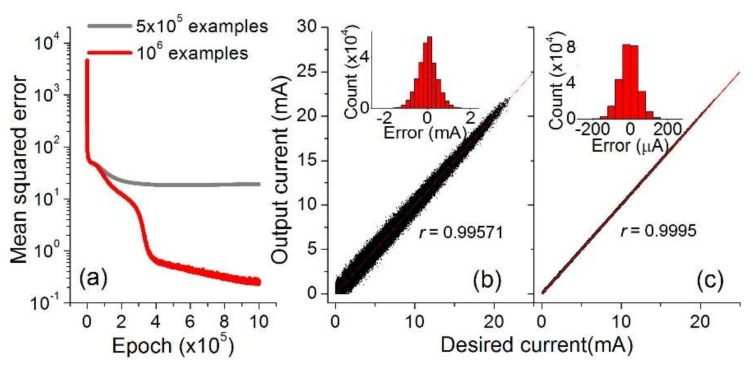
(**a**) Training the network (2,500 units in each of two hidden layers) with 500,000 and 1,000,000 examples for Type B switch. The capability of response inference is shown for the network trained with (**b**) 500,000 and (**c**) 1,000,000 examples. The insets address the distribution of inference-error.

The correlation coefficient *r* for each case was also evaluated as another measure of success of training, which is given by $r=cov\left(I_{out}-I_{cor}\right)/\sqrt{var\left(I_{out}\right)\cdot var\left(I_{cor}\right)}$, where *cov* and *var* denote a covariance and variance, respectively. The correlation coefficient is asymptotic to 1 when the inference error tends to zero, and thereby *r* = 1 implies zero error (perfect match). The calculated *r* for each case is written in Figure 2. The failure of training for the network with 2,500 units in each of the two hidden layers is due to overfitting (see orange curves in Figure 2c,d). Although the network is given sufficient complexity (a large number of units and hidden layers) to learn the complex input pattern, insufficient training examples lead to faulty training as shown in the orange curves (Figure 2c,d). Overfitting could be avoided by training with a larger training dataset (here 1,000,000 examples for Type B switch) as shown in Figure 3a. The inference-error for the overfitting case is detailed in Figure 3b which represents a substantial discrepancy between the inferred and desired outputs, the extent to which the RMSE reaches 438.2 μA (*r* = 0.99571). The error statistics are plotted in the inset. In contrast, a remarkable reduction in inference-error is identified for the non-overfitting case (Figure 3c) whose RMSE is lowered down to 49.2 μA (*r* = 0.9995).

**Figure 4:**
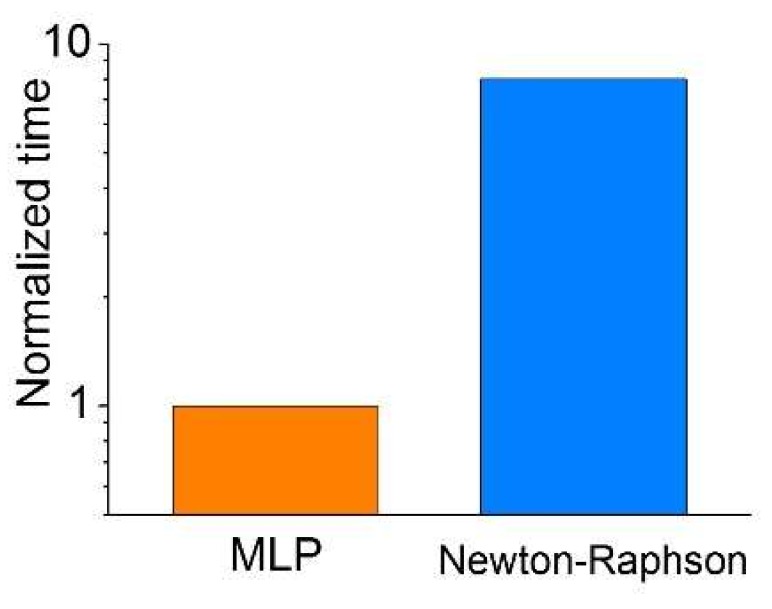
Comparison of run time for the proposed method and Newton-Raphson method.

Finally, we compared the time-efficiency of the proposed method with the conventional Newton-Raphson method [22]. The run time of a 10 × 9 resistance array calculation was measured for both methods using the same computer. The result shown in Figure 4 ensures an acceleration in calculation by approximately 8 times, identifying a feasible benefit of fast calculation from the proposed method.

## 6. Conclusions

A fully connected feed-forward network with different structures (depth and the number of activation units) was successfully trained to infer the current response of a random crossbar array to a randomly applied voltage array. This work first verifies the capability of ANN to capture the highly nonlinear input-output relationship of a crossbar array model system. Secondly, MLP for supervised learning provides a means of real-valued array inference beyond the classification of input patterns. Thirdly, this work offers a distinct view of crossbar array evaluation—a numerical solution of a number of simultaneous equations can be avoided at the expense of a few steps of matrix-vector product for inference. However, training the network and preparing datasets can be expensive, depending on the network hyper-parameters and model crossbar array size. Thus, we leave this efficiency issue open for the moment.

## Figures and Tables

**Table 1:** Parameters of model switch.

Heading	Type A	Type B
*R*_HRS_ (Ω)	10^8^ × e^−*V*^	10^5^ × e^−*V*^
*R*_LRS_ (Ω)	10k	1k
*R*_HRS_/*R*_LRS_ at 1 V	3679	36.79
